# Book Review: Another Science Is Possible

**DOI:** 10.3389/fpsyg.2018.00455

**Published:** 2018-04-04

**Authors:** Jose D. Perezgonzalez, Dolores Frías-Navarro, Juan Pascual-Llobell

**Affiliations:** ^1^Business School, Massey University, Palmerston North, New Zealand; ^2^Department of Methodology of the Behavioral Sciences, Universitat de València, Valencia, Spain

**Keywords:** science, society, slow science, philosophy, error statistics, Bayesian inference

The philosopher of science Isabelle Stengers provides some food for thought regarding both the way we are doing science and the need for an alternative approach likened to the slow movement in other spheres of life.

The title of the book already promises a dialectical contrast between contemporary and another form of science, and between fast and slow science. The remainder of the book does not disappoint in such strategy. Indeed, Stengers does a good job in focusing on different contrasts in the five main chapters comprising the book (the sixth and last chapter mostly wraps up what had been said before).

Stengers's chief contrast is between Science and Society: Science pursuant of knowledge, of facts, of right answers to specific problems by specialist people; Society as the net beneficiary of Science's work but also as a mass which confuses facts and values because it often lacks the scientific literacy to spot the difference (Ch. 1). Stengers argues against Science's technocratic mindset and in favor of Society's democracy, which needs from Science contextualized answers to its social concerns and the cultivation of a public intelligence of connoisseurs.

Stengers next uses gender in lieu of “marked” scientists to identify a second contrast, that between “hard” (or “sound”) sciences and “soft” sciences (Ch. 2). For Stengers, Science is mostly about mimicking the hard sciences, about scientists having the “right stuff,” focused on facts and laboratory objectivity, mobilized in serving industrial interests. “Marked” scientists are those who deviate from above ideal to become concerned with social matters, either historically (women) or contemporarily (youth avoiding the hard sciences, and scientists inclined toward “soft” matters).

As the book progresses, Stengers tackles contemporary research autonomy and evaluation, identified as “fast” science and intimately correlated with competitive evaluation, publication in high-impact-factor journals, inbreeding review by peers, and industrial capture of financial research resources (Ch. 3). By contrast, Stengers calls for a contested evaluation, a slow-down of publications and peer-review, and a reclaiming of social interdependency as a definition for scientific excellence.

She follows such call by explicitly linking to the 2010 “Slow Science Manifesto” (The Slow Science Academy, 2010), which she contrasts against her own idea of slow science (Ch. 4). For Stengers, slow science is not about returning to the (fast science) golden era where scientists were autonomous and respected, but about creating a collective awareness and appreciation for Society among scientists (i.e., for them to “become civilized”).

Finally, Stengers brings her slow science plea to academia, and tasks the university with creating such a future for slow science, of complementing the reliability of the laboratory with the reliability of the context of application, and of bringing value to facts (Ch. 5).

Ultimately, the book delivers a different idea than its title promised. It is not about another science, but about contemporary science communicated and applied differently, more attuned to Society's milieu. Nor is the book in line with the Berlin manifesto for slow science but about Science slowing down so that it can be successful in the above form of attunement.

The book also has two small drawbacks. One is stylistic: Stengers did not apply to her own philosophy her criticisms of what Science is doing, insofar her book has not left her own “Ivory Tower” of circumloquacious writing and conceptual detours ending in cul-de-sacs, possibly highly appreciated by her peers but taxing other readers unnecessarily (indeed, about 80% of the text could be safely dismissed without affecting the main ideas in the book).

The second drawback is implementation: Stengers takes herself out of the fight by book's end, in a way reminiscent of a criticism she had earlier laid onto scientists, as it seems she equally “[does] not feel there is an option at all” (p. 110). Her calls are, thus, “only suggestions…to try to activate the imagination” (p. 124), “a little derisory” (p. 142), “a philosopher['s]…dream, for such a counterfactual story” (p. 144).

And yet, all the time we have spent reading (and re-reading) Stengers's book, we kept wondering about a related contrast, that of the statistics wars between frequentists and Bayesians. Indeed, not long ago, another philosopher of science, Deborah Mayo, lashed out against Bayesians in what parallels a defense—by Mayo—of current practices of laboratory research for “warranting a scientific research claim, or learning about a substantive phenomenon of interest” (Mayo, 2017a). She correctly argued that “in an adequate account [of severity testing], the improbability of a claim must be distinguished from its having been poorly tested. (You need to be able to say things like, ‘it's plausible, but that's a lousy test of it.')” (Mayo, 2017b). The relevance of Mayo's stance in favor of research objectivity and severe testing needs to be defended. However, Mayo did not tackle the alternative consequence to her claim, an alternative which underlies Stengers's ideas: that you also need to be able to say things like, “it may have been reliably tested, but its social reliability is nonetheless lousy.”

This contrast between claims that need to be severely tested (e.g., Mayo and Spanos, 2010) and applications that need to be reliably assessed in the wider context of application thus suggests a method for scientists to move from the laboratory to the social milieu: Bayesian inference (e.g., Kruschke, 2011). With a Bayesian inference built upon error statistics, Stengers's contextual reliability would combine with scientific reliability to respond to the important question regarding the (subjective) value of an (objective) fact, both before implementation as well as throughout the life-cycle of those solutions already implemented. The initial advantage of this method rests on the preference scientists already have toward quantification and formulation, yet forces them to further consider those social “matters of concern” that may escape them in their daily scientific milieu. This method may, thus, provide substance to Stengers's slow science manifesto and a practical solution to its implementation.

## Author contributions

JP acquired the funding, and initiated and drafted the review. DF-N, JP-L and JP revised and edited the manuscript. All authors approved the final version of the manuscript for submission.

### Conflict of interest statement

The authors declare that the research was conducted in the absence of any commercial or financial relationships that could be construed as a potential conflict of interest.
